# The *HMGA1* Pseudogene *7* Induces miR-483 and miR-675 Upregulation by Activating *Egr1* through a ceRNA Mechanism

**DOI:** 10.3390/genes8110330

**Published:** 2017-11-17

**Authors:** Marco De Martino, Giuseppe Palma, Amalia Azzariti, Claudio Arra, Alfredo Fusco, Francesco Esposito

**Affiliations:** 1 Istituto di Endocrinologia ed Oncologia Sperimentale del CNR c/o Dipartimento di Medicina Molecolare e Biotecnologie Mediche, Scuola di Medicina e Chirurgia di Napoli, Università degli Studi di Napoli “Federico II”, via Pansini, 5, 80131 Naples, Italy; marco.demartino2@unina.it (M.D.M.); alfusco@unina.it (A.F.); 2 Istituto Nazionale dei Tumori, Fondazione Pascale, via Mariano Semmola, 52, 80131 Naples, Italy; palma.giuseppe@icloud.com (G.P.); c.arra@libero.it (C.A.); 3 IRCCS Istituto Tumori Giovanni Paolo II, Viale O. Flacco, 65, 70124 Bari, Italy; a.azzariti@oncologico.bari.it

**Keywords:** *HMGA1* pseudogenes, *HMGA1P7*, micro RNA, ceRNA

## Abstract

Several studies have established that pseudogene mRNAs can work as competing endogenous RNAs and, when deregulated, play a key role in the onset of human neoplasias. Recently, we have isolated two *HMGA1* pseudogenes, *HMGA1P6* and *HMGA1P7*. These pseudogenes have a critical role in cancer progression, acting as micro RNA (miRNA) sponges for *HMGA1* and other cancer-related genes. *HMGA1* pseudogenes were found overexpressed in several human carcinomas, and their expression levels positively correlate with an advanced cancer stage and a poor prognosis. In order to investigate the molecular alterations following *HMGA1* pseudogene *7* overexpression, we carried out miRNA sequencing analysis on *HMGA1P7* overexpressing mouse embryonic fibroblasts. Intriguingly, the most upregulated miRNAs were miR-483 and miR-675 that have been described as key regulators in cancer progression. Here, we report that *HMGA1P7* upregulates miR-483 and miR-675 through a competing endogenous RNA mechanism with *Egr1*, a transcriptional factor that positively regulates miR-483 and miR-675 expression.

## 1. Introduction

Pseudogenes are classified into two major groups: processed and unprocessed pseudogenes [1,2]. Unprocessed pseudogenes are duplicated pseudogenes that take in introns and, occasionally, upstream regulatory elements since they are generated by gene duplication [1,2]. Indeed, a small subclass of unprocessed pseudogenes, called unitary pseudogenes, take place when protein-coding genes acquire mutations and lose their coding potential [1,2]. The other main group is represented by the processed pseudogenes, which are derived from an ancestral retrotransposition of a parental gene mRNA. They, therefore, are limited in structure to a single exon [1,2]. Generally, the distribution of pseudogenes is entirely random, in fact, duplicated and processed pseudogenes are found in the same or on different chromosome of their parental genes [1,2].

Pseudogenes have been thought for long time as relict copies of protein-coding genes unable to yield a functional protein product as their parental genes because of gene truncation or mutation [1,2].

However, it has recently emerged that several pseudogenes are functional since they are transcribed and some of them are translated into proteins, which frequently play their function on parental genes by competing for RNA-binding proteins or the translation machinery [3,4,5]. More recently, many studies have established alternative ways by which pseudogene transcripts can regulate gene expression.

For example, pseudogenes can strongly alter the expression of their parental and other genes by acting as a microRNA (miRNA) sponges [6,7]. Moreover, when a pseudogene is transcribed in antisense orientation with respect to its parental gene, pseudogene transcript can negatively regulate parental gene by hybridizing to its transcript or epigenetically targeting its promoter [8]. Indeed, siRNA can be produced by processing pseudogene mRNAs, which can silence genes by interfering with their transcription [9].

We have recently described *HMGA1P6* and *HMGA1P7* pseudogenes, likely derived from the retrotransposition of *HMGA1* oncogene. HMGA1 belongs to the High Mobility Group A protein family, architectural proteins that are overexpressed in all the malignant tumours and their overexpression is correlated with a poor patient survival [10,11]. Indeed, its overexpression induces in vitro cell transformation [12,13] and benign and malignant neoplasias in mice [11]. *HMGA1* non-coding pseudogenes (*HMGA1Ps*), *HMGA1P6* and *HMGA1P7*, are two processed pseudogenes that show conserved seed matches for miRNAs targeting the *HMGA1* gene and other cancer-associated genes [1,14,15,16]. Following their overexpression, *HMGA1Ps* act as competitive endogenous RNAs (ceRNAs) and enhance HMGA1 protein levels, thereby inhibiting the repression of HMGA1 protein synthesis exerted by miRNAs [1,14,15,16]. Furthermore, *HMGA1Ps* overexpression equally upregulates the expression of other cancer-related genes, since the *HMGA1Ps* transcripts take in additional seed sequences for miRNAs capable of targeting many oncogenes [1,14,15,16]. Finally, a direct correlation among *HMGA1*, *HMGA1P6*, and *HMGA1P7* expression in a group of human thyroid, ovary, endometrial, and pituitary tumours has been shown [1,14,15,16,17].

To investigate new mechanisms by which *HMGA1Ps* act as oncogenes, we studied the miRNAs profile expression in mouse embryonic fibroblasts (MEFs) deriving from *HMGA1P7* transgenic mice in comparison with the wild-type (WT) ones, using a miRNA sequencing (miRNA-seq) technique. Thanks to this analysis, we obtained a set of miRNAs up- or down-regulated in *HMGA1P7* overexpressing MEFs compared with WT cells. Among them, we focused our attention on two of the most overexpressed miRNAs: miR-483 and miR-675. Intriguingly, it has been extensively demonstrated that miR-483 and miR-675 are two oncomiRs since they have been found overexpressed in many tumours such as prostate [18], gastric [19], Wilms’ [20], adrenocortical [21], esophageal [22], breast [23], colon [24], and lung tumours [25]. Here, we demonstrate that *HMGA1P7* upregulates miR-483 and miR-675 through the activation of *Egr1* by a ceRNA mechanism.

## 2. Materials and Methods

### 2.1. Cell Culture and Transfections

MEFs and NIH3T3 were cultivated in DMEM complemented respectively with 10% foetal calf serum (Thermo Fisher Scientific Inc., Waltham, MA, USA) and calf serum (Thermo Fisher Scientific Inc., Waltham, MA, USA), glutamine, and antibiotics. MycoAlert (Lonza, Walkersville, MD, USA) was regularly used to check that cells were not infected by mycoplasma. Lipofectamine plus reagent was used to transfect the cells (Thermo Fisher Scientific Inc., Waltham, MA, USA) according to the manufacturer’s instructions. Medium containing Geneticin (Sigma, St. Louis, MO, USA) was used to select transfected cells. GFP signal was used to assess transfection efficiency for each experiment. To inhibit Dicer expression, short interfering RNAs and corresponding scramble short interfering RNAs were used as suggested by the manufacturer (RIBOXX, Radebeul, Germany).

### 2.2. Bioinformatic Analysis Procedure for MicroRNA Analysis

Reads (sequence and quality) obtained with the SOLiD sequencing have been mapped in Color Space using the Lifescope ver. 2.5.1 software “small RNA” pipeline. Target databases were the reference genome GRCm38/mm10 (Dec 2011) and the dataset of mature sequences + precursors miRbase version 20 (June 2013). Matches with repetitive regions of the human genome such as short interspersed nuclear elements (SINEs), long interspersed nuclear elements (LINEs), small nucleolar RNAs (snoRNAs), piwi-interacting RNAs (piRNAs), tRNAs, rRNAs were eliminated by the mapping results using an “ad hoc” created database starting from Rfam sequences and the “small RNA” pipeline.

The known miRNA count has been analysed with the Bioconductor statistical library edgeR for R 64 bit version, version 3.02, and Genomnia (Bresso, Italy) proprietary analytical parameters.

*P*-value, uncorrected or corrected (“adj.P.Val”) for multiple comparisons with the Benjamini and Hochberg method to control the False Discovery Rate (FDR), was used. The significance threshold considered for the FDR in these comparisons was 0.05. The data were deposited in European Nucleotide Archive with the Project ID PRJEB23361.

### 2.3. Mouse Embryo and Tissue Samples

The experiments performed on mouse embryos and tissues were carried out in accordance with the project “Ruolo degli pseudogene di HMGA1 nel cancro” Cod. 893/2013 approved by Italian Health Ministry on 13/05/2013; the methods and experiments were carried out in accordance with the approved guidelines by the Ministero della Salute.

### 2.4. RNA Extraction and Quantitative Reverse Transcription PCR

Total RNA was isolated from cells or tissues with TRIsure (Bioline, London, UK) according to the manufacturer’s instructions. For quantitative mRNA detection, we reverse transcribed total RNA from samples by utilizing QuantiTect Reverse Transcription Kit (Qiagen, Hilden, Germany), and then Real-time PCR was performed by using Power SYBR Green PCR Master Mix (Bio-Rad) and the following primers: mG6pd-Fw5′ -cagcggcaactaaactcaga-3′, mG6pd-Rev 5′ -ttccctcaggatcccacac-3′, mEgr1 Fw 5′ -cctatgagcacctgaccaca-3′, mEgr1 Rev 5′ -tcgtttggctgggataactc-3′, mDicer Fw 5′ -ggtccgatggttctggaag-3′, mDicer Rev 5′ -gcaaagcagggcttttca-3′. For miRNA detection, total RNA from cells or tissues was reverse-transcribed using miScript Reverse Transcription Kit (Qiagen, Hilden, Germany). Real-Time PCR was performed following miScript System Kits (Qiagen, Hilden, Germany) instructions [26]. Real-Time PCR reactions contained miScript Primer Sets (Qiagen, Hilden, Germany), specific for Mm-mir-483-5p 5′-aagacgggagaagagaagggag -3′, Mm-mir-483-3p 5′-ucacuccuccccucccgucuu-3′, Mm-mir-675-5p 5′-uggugcggaaagggcccacagu-3′, Mm-mir-21-3p 5′-caacagcagucgaugggcuguc-3′, Mm-mir-187-3p 5′-ucgugucuuguguugcagccgg-3′, Mm-mir-214 5′-acagcaggcacagacaggcagu-3′, Mm-mir-761 5′-gcagcagggugaaacugacaca-3′. U6 (cod. MS00033740) (used to normalize RNA levels). The 2^−∆∆CT^ formula was used to calculate the differential gene expression and is described elsewhere [27].

### 2.5. miRNA Oligonucleotides and Transfection

The transfections of miRNA oligonucleotides were performed by transfecting the cells with 50 nmol/ml of miRNA precursors or with a control no-targeting scrambled oligonucleotides (Thermo Fisher Scientific Inc., Waltham, MA, USA) using siPORT neoFX Transfection Agent (Thermo Fisher Scientific Inc., Waltham, MA, USA).

### 2.6. Protein Extraction, Western Blotting and Antibodies

Protein extraction and Western blotting were performed as previously described [28,29]. The primary antibodies used were anti-Egr1 (ab191441) from Abcam (Cambridge, UK); anti-Gapdh (sc-32233) and anti-Vinculin (sc-7649) from Santa Cruz Biotechnology (Dallas, TX, USA); anti-α-Tubulin (t6074) from Sigma (St. Louis, MO, USA). Blots were visualized by using the Western blotting detection reagents (Thermo Fisher Scientific Inc., Waltham, MA, USA).

### 2.7. Statistical Analysis

Statistical analyses were performed using GraphPad Prism, GraphPad Software, Inc. (La Jolla, CA, USA). The comparison between two groups of experiments was carried out using Student’s *t* test. Results are reported as means ± SD and differences were considered to be significant with *p* < 0.05.

## 3. Results

### 3.1. miRNA-seq on HMGA1P7 Transgenic MEFs

**Figure 1:**
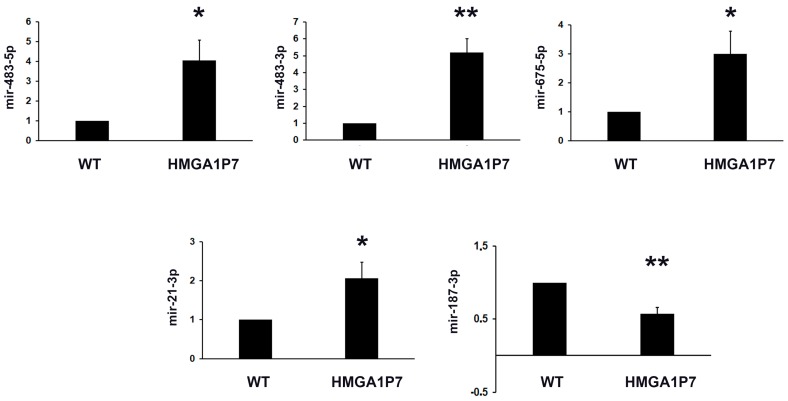
Validation of miRNA-seq data by qRT-PCR. qRT-PCR analysis of miR-483, miR-483-3p, miR-675-5p, miR-21-3p, and miR-187-3p in mouse embryonic fibroblasts (MEFs) from *HMGA1P7* transgenic mice compared to wild-type (WT), set equal to 1. The results are reported as the mean of values. The error bars represent mean ± SD; *: *p* < 0.05, **: *p* < 0.01 (*t* test).

First, we evaluated the whole miRNome of WT and *HMGA1P7* transgenic MEFs, obtained from mice modified to overexpress *HMGA1 pseudogene 7*, by miRNA-seq analyses to identify the effects of *HMGA1P7* upregulation on miRNAs expression. To this purpose, the total population of miRNAs isolated from WT- and *HMGA1P7*-MEFs was sequenced. Then, through biostatistical analysis, we acquired a list of miRNAs that were differentially expressed (FDR adjusted *p*-value of 0.05) between WT and *HMGA1P7* transgenic MEFs (Supplementary Table S1). The next step was to confirm the results obtained by miRNA-seq analyses, investigating five deregulated miRNAs by qRT-PCR. The results reported in Figure 1 confirm the overexpression of miR-483-5p, miR-483-3p, miR-675-5p, miR-21-3p, and the downregulation of miR-187-3p in *HMGA1P7* overexpressing MEFs in comparison with the WT ones. Interestingly, these miRNAs have been associated to human cancers (such as colon, adrenocortical, oral, lung, hepatocellular, prostate, ovarian) and could be considered possible therapeutic targets [24,30,31,32,33,34,35,36,37,38,39].

### 3.2. miR-483-5p and miR-675-5p are Upregulated in HMGA1P7 Mouse Tissues

**Figure 2:**
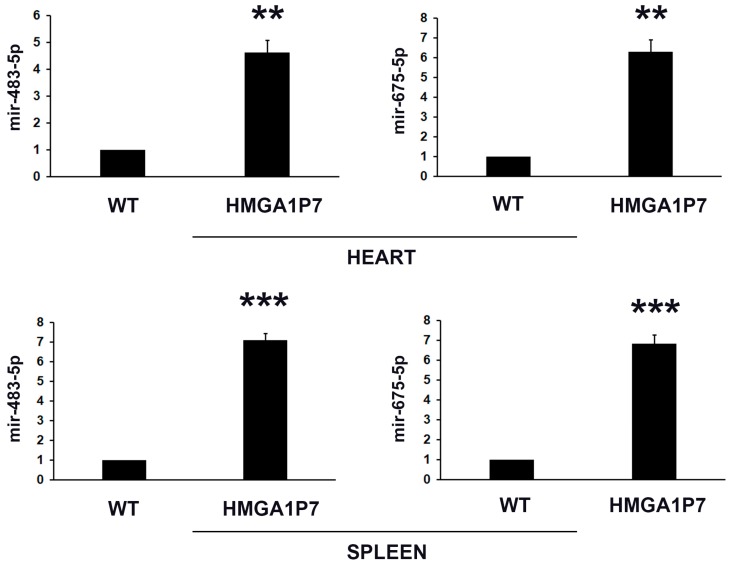
mir-483-5p and mir-675-5p are overexpressed in *HMGA1P7* transgenic mouse tissues. qRT-PCR analysis of mir-483-5p and mir-675-5p from hearts (upper panel) and spleens (lower panel) explanted from WT and *HMGA1P7* transgenic mice. The results are reported as the mean of values. The error bars represent mean ± SD; *: *p* < 0.05, **: *p* < 0.01, ***: *p* < 0.001 (*t* test).

Then, we converged our studies on the miR-483-5p and miR-675-5p, which showed the most upregulated fold-change in *HMGA1P7* overexpressing MEFs by miRNA-seq analyses. Moreover, their expression has been recurrently found deregulated in human cancer [24,30,31,34]. As expected from previous data, qRT-PCR analysis showed that miR-483-5p and miR-675-5p were also upregulated in heart and spleen from *HMGA1P7* adult transgenic mice (Figure 2).

### 3.3. HMGA1P7 Pseudogene Sustains miR-483-5p and miR-675-5p Expression via a ceRNA Mechanism with Egr1

**Figure 3:**
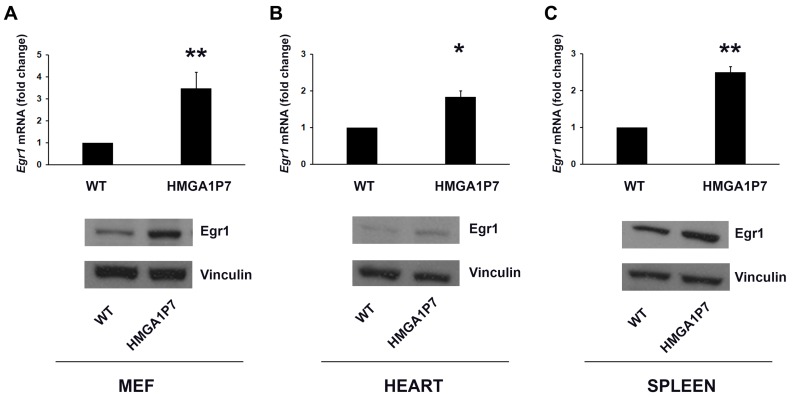
Egr1 is overexpressed in *HMGA1P7* transgenic mouse tissues. qRT-PCR and Western Blot analyses of Egr1 levels in MEFs (**A**), hearts (**B**), and spleens (**C**) derived from WT and *HMGA1P7* transgenic mice. The results are reported as the mean of values. The error bars represent mean ± SD; *: *p* < 0.05, **: *p* < 0.01 (*t* test).

**Figure 4:**
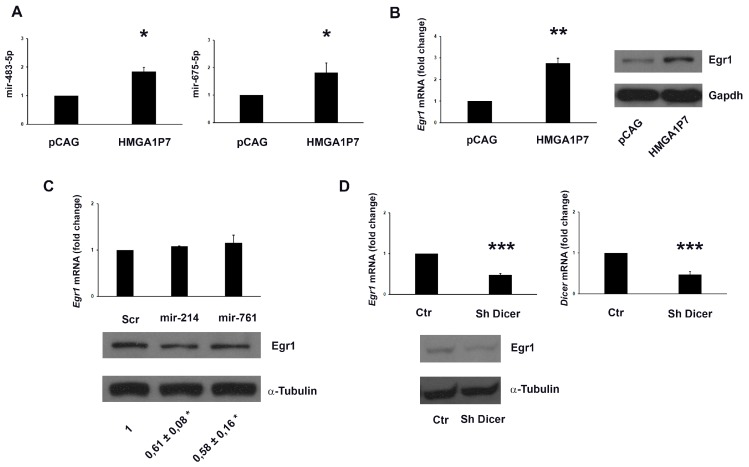
*Egr1* is positively regulated by *HMGA1P7* through ceRNA connection. (**A**) qRT-PCR analysis of mir-483-5p and mir-675-3p from control and overexpressing NIH3T3 cells. (**B**) Left panel, qRT-PCR analysis of *Egr1* from control and *HMGA1P7* overexpressing NIH3T3 cells. Right Panel, Western blot analysis of Egr1 from control and *HMGA1P7* overexpressing NIH3T3 cells. (**C**) Upper panel, qRT-PCR analysis of *Egr1* mRNA from the NIH3T3 cells transfected with scrambled-oligonucleotide, miR-214, miR-761. Lower panel, a representative Western blot analysis of Egr1 in the same samples as in the upper panel. Densitometric analysis of Western blot assays for Egr1 is also reported. (**D**) Upper panel, *Egr1* and *Dicer* mRNA levels 24 h after the transfection of scrambled oligonucleotide or siRNA-Dicer in *HMGA1P7* NIH3T3 overexpressing cells. Lower Panel, Western blot analysis of Egr1 in the same samples as in the upper panels. The results are reported as the mean of values. The error bars represent mean ± SD; *: *p* < 0.05, **: *p* < 0.01, ***: *p* < 0.001 (*t* test).

It has been reported that *early growth response protein 1* (*Egr1*) controls the expression of *H19* [40] and *Igf2* [41] and that, intriguingly, miR-483 is located within the second intron of *Igf2* gene [42] and miR-675 is encoded by the first exon of *H19* gene [40]. These results suggest that Egr1 could regulate miR-483 and miR-675 expression by upregulating *H19* and *Igf2* genes. Since we found the upregulation of *Egr1* in *HMGA1P7* overexpressing MEFs performing RNA-seq analysis [1], we tested whether it could be responsible for the miR-483-5p and miR-675-5p upregulation. As shown in Figure 3, we found Egr1 overexpressed in *HMGA1P7* transgenic MEFs and tissues analyzed by qRT-PCR and western blot analyses. Moreover, as expected from previous results, qRT-PCR showed upregulation of miR-483-5p and miR-675-5p following *HMGA1P7* pseudogene overexpression in NIH3T3 cells (Figure 4A). Further analyses exhibited again the associated Egr1 upregulation, at mRNA and protein levels, in *HMGA1P7*-transfected NIH3T3 cells (Figure 4B). Taken together, these data deeply endorse the assumption that *HMGA1P7* could act as ceRNA for *Egr1*, which in turn upregulates miR-483-5p and miR-675-5p.

### 3.4. HMGA1P7 Works as Decoy for Egr1 Targeting miRNAs

To check whether the influence of the *HMGA1P7* pseudogene on *Egr1* expression is related to sharing targeting-miRNAs, we tested the ability of *HMGA1P7*-targeting miRNAs to bind to *Egr1*. To this intention, we transfected miR-214 and miR-761 (already demonstrated to target *HMGA1P7* [1]) into NIH3T3 cells, and evaluated Egr1 mRNA and protein levels. As presented in Figure 4C, lower panel, the transfection of the *HMGA1P7*-targeting miRNAs produced a significant reduction of Egr1 protein levels. qRT-PCR analysis showed that the *Egr1* downregulation occurs only at translational level after the transfection of the *HMGA1P7*-targeting miRNAs (Figure 4C, upper panel). Finally, we demonstrated that the overexpression of Egr1, generated by upregulation of *HMGA1P7*, was reduced in Dicer-knockdown cells (Figure 4D) then supporting the idea that *HMGA1P7* and *Egr1* are modulated by the same miRNA-mediated post-transcriptional regulation. Indeed, the knocking down of the miRNA-processing enzyme Dicer abolished the effects of miRNAs on their target genes, reducing the levels of mature miRNAs. These evidences are coherent with the conclusion that *HMGA1P7* requires mature miRNAs to regulate *Egr1* levels and then upregulates miR-483-5p and miR-675-5p.

## 4. Discussion

Pseudogenes are genomic loci that look like protein-encoding genes, but until now have been considered biologically insignificant because they do not generate working proteins. However, according to recent studies, pseudogenes are attracting considerable research interest thanks to their recognized function. To date, very few pseudogenes have been functionally characterized. Among these, *HMGA1P6* and *HMGA1P7* pseudogenes have caught our attention. In fact, they work as sponges for several miRNAs so that their overexpression defends the *HMGA* gene family and other oncogenes from miRNAs negative regulation. In the present study, we identified miRNAs differentially expressed in MEFs overexpressing *HMGA1P7* by miRNA-seq analysis. Looking at the results, we found that the levels of several miRNAs were modulated by *HMGA1P7* expression. Noteworthy, most of them are known as oncomiRs involved in cancer promotion and progression. For example, miR-483-5p has been identified as predictor of poor prognosis in adrenocortical cancer and it has been demonstrated to promote invasion and metastasis of lung adenocarcinoma by targeting RhoGDI1 and ALCAM [30,31]. Moreover, it has been reported that overexpression of miR-483-3p overcomes miR-145/TP53 pro-apoptotic loop in hepatocellular carcinoma and that it mediates its oncofunction by suppressing DLC-1 in colorectal cancer [32,33]. Furthermore, miR-675-5p has been found overexpressed in metastatic colon cancer cells and it is able to induce resistance to 1,25(OH)2D3 by targeting VDR [24,34]. In addition, also miR-21-3p has been described as oncomiR since it promotes oral cancer metastasis and colorectal cancers [35,36], and its silencing resulted in important reduction of ovarian and prostate cancer cell proliferation and invasion [37]. Finally, in the opposite way, miR-187-3p has been reported as oncosuppressor because of its inhibitory action on metastasis and epithelial-mesenchymal transition of hepatocellular carcinoma and non-small cell lung cancer [38,39]. In this study, we focused on miR-483-5p and miR-675-5p since they are involved in carcinogenesis and they belong to the *H19*/*Igf2* locus, which has been already linked to *HMGA1P7* ceRNA network [1,40,42]. Intriguingly, *H19*/*Igf2* locus is under transcriptional control of Egr1 [40,41], that we have already found upregulated in *HMGA1P7* transgenic MEFs by mRNA-seq analysis [1]. Indeed, Egr1 controls the transcription of *H19* and *Igf2* whose mRNAs maturation generates miR-483-5p and miR-675-5p [40,41]. Here, we demonstrate that *HMGA1P7* overexpression enhances *Egr1* levels suppressing its mRNA inhibition by miRNAs that target *HMGA1P7* gene. Consequently, *H19* and *Igf2* mRNAs increase and, with them, so do miR-483-5p and miR-675-5p amounts. In conclusion, the results described here reinforce the already known oncogenic role of the *HMGA1P7* pseudogene as result of ceRNA mechanisms that enhance the expression of cancer-related genes. Interestingly, our preliminary results support the oncogenic role of *HMGA1P7 in vivo* since malignant haematological neoplasias develop in *HMGA1P7* transgenic mice (manuscript in preparation). Therefore, the results presented here envisage the possibility of an innovative cancer therapy based on the suppression of *HMGA1 pseudogenes* expression and/or the overexpression of miRNAs involved in their ceRNA pathways.

## Supplementary Materials

The following are available online at www.mdpi.com/2073-4425/8/11/330/s1.
