# Supplementary material for: The HMGA1 Pseudogene 7 Induces miR-483 and miR-675 Upregulation by Activating Egr1 through a ceRNA Mechanism
Source: Genes (Basel). 2017 Nov 17;8(11):330. doi: 10.3390/genes8110330 (PMC5704243; doi:10.3390/genes8110330)
Supplement: Supplementary file 1 [file genes-08-00330-s001.pdf]

Supplementary Material

Supplementary Table 1. List of miRNAs deregulated in HMGA1P7 overexpressing MEFs vs WT MEFs. In the Differential Expression Table the Fold Change is referred to the HMGA1P7 overexpressing MEFs compared to the Control; the "logCPM" column reports the total concentration of a given microRNA, in Count Per Million, as a mean between the two groups involved in the comparison; the "logFC" column instead reports the logarithm of Fold Change for the counts of the two groups which are being compared. A negative logFC implies that the expression of a given microRNA is lower in the reference group. Following we have two columns which report the P-value, uncorrected or corrected ("adj.P.Val") for multiple comparisons with the Benjamini and Hochberg method to control the False Discovery Rate (FDR). The significativity threshold considered for the FDR in these comparisons was 0.05.

**miRNA_ID**	**logFC**	**logCPM**	**PValue**	**FDR**
mmu-miR-7082-5p	5.31	4.75	0.00000	0.00000
mmu-miR-6906-5p	5.30	1.07	0.00000	0.00000
mmu-miR-675-5p	3.67	2.30	0.00000	0.00024
mmu-miR-483-3p	3.07	5.58	0.00005	0.00472
mmu-miR-483-5p	2.89	6.16	0.00015	0.00952
mmu-miR-675-3p	2.44	5.06	0.00005	0.00472
mmu-miR-1949	2.24	5.48	0.00008	0.00633
mmu-miR-2137	2.21	9.43	0.00000	0.00000
mmu-miR-21a-3p	1.44	8.72	0.00016	0.00952
mmu-miR-187-3p	-1.54	6.16	0.00067	0.03519
